# Three-Dimensional Assessment of Mandibular Morphology Across Vertical Facial Growth Patterns Using Cone-Beam Computed Tomography: A Cross-Sectional Study

**DOI:** 10.7759/cureus.84895

**Published:** 2025-05-27

**Authors:** R. Rajesh, Shubham Gupta, Soi Chakraborty, Swapnali S Patil, Arun Chauhan, Anurag Mukherjee, Seema Gupta

**Affiliations:** 1 Department of Orthodontics, Kothiwal Dental College and Research Centre, Moradabad, IND; 2 Department of Orthodontics, Kusum Devi Sunderlal Dugar Jain Dental College and Hospital, Kolkata, IND; 3 Department of Public Health Dentistry, D Y Patil Dental School, Pune, IND

**Keywords:** cone-beam computed tomography, facial, mandibular, morphology, patterns

## Abstract

Introduction: Mandibular morphology exhibits significant variation across vertical facial growth patterns. A clear understanding of these variations is essential for precise orthodontic diagnosis, treatment planning, and the prediction of growth trends. This study aimed to compare the mandibular morphological characteristics among individuals with normodivergent, hypodivergent, and hyperdivergent growth patterns using cone-beam computed tomography (CBCT), focusing on parameters such as condylar dimensions, ramus morphology, symphysis measurements, and mandibular notch features.

Materials and methods: A cross-sectional observational study was conducted at the Department of Orthodontics and Dentofacial Orthopaedics. Based on Jarabak’s ratio, the 27 subjects (aged 18-36 years) were categorized into three groups (normodivergent, hypodivergent, and hyperdivergent). CBCT scans were acquired using standard protocols and were analyzed. Mandibular parameters, including condylar width, length, height, shape, coronoid process, mandibular notch morphology, ramus height and width, and symphysis height and thickness were measured. All the statistical analyses were performed. One-way analysis of variance (ANOVA) and Tukey’s post-hoc test were used to assess intergroup differences, and Spearman’s correlation was used to analyze the relationship between the mandibular notch type and other measurements. Statistical significance was set at p < 0.05.

Results: Type 4 condylar shape and Type 1 coronoid process were the most prevalent but showed no significant association with growth pattern. Significant differences were observed in ramus width (p = 0.017) and symphysis thickness (p = 0.036), particularly between the hypodivergent and hyperdivergent groups. The hyperdivergent group exhibited taller condyles, narrower rami, and increased symphysis height, while the hypodivergent group showed thicker symphyses and broader rami. Correlation analysis revealed weak and inconsistent relationships between the mandibular notch type and mandibular measurements, with the strongest correlation noted between the notch type and symphysis height (r = -0.52).

Conclusion: Mandibular morphology demonstrated measurable variation across vertical facial growth patterns. While some dimensions, such as ramus width and symphysis thickness, differed significantly, mandibular notch morphology appeared largely independent of other anatomical features.

## Introduction

Craniofacial growth is a multifaceted biological process shaped by the interplay between genetic, developmental, and environmental factors [1]. It occurs primarily at specific growth sites and sutures that undergo dynamic remodeling influenced by both intrinsic programming and external stimuli [1]. A thorough understanding of these growth patterns is fundamental in orthodontics and maxillofacial diagnosis, enabling clinicians to distinguish between normal and aberrant development and plan treatment accordingly.

A key component of the craniofacial complex is the mandible. As the largest and most functionally significant bone in the facial skeleton, the mandible plays a crucial role in mastication, speech, airway maintenance, and overall facial aesthetics [2]. Its morphology is not uniform across individuals; rather, it varies considerably depending on skeletal and dental patterns, especially vertical facial proportions [3]. These proportions are essential in establishing facial harmony and balance and have been studied extensively in relation to orthodontic diagnosis and treatment. The mandible exhibiting a vertical growth pattern is usually correlated with a symphysis characterized by considerable height, reduced depth, an elevated ratio, an increased mandibular plane angle, a reduction in ramus height and width, a lesser mandibular depth, and a decreased mandibular arc angle when compared to the mandible displaying a horizontal growth pattern [4].

Facial types are typically classified based on skeletal divergence into normodivergent, hypodivergent, and hyperdivergent patterns [4]. Each growth pattern exhibited distinct mandibular features. For instance, hyperdivergent individuals tend to have a backwardly inclined condylar head, pronounced antegonial notch, elongated anterior facial height, and retroclined symphysis [4,5]. Conversely, hypodivergent individuals usually display a forwardly inclined condyle, flatter mandibular border, reduced anterior facial height, and protrusive symphyseal inclination [4,6].

Although widely used, traditional two-dimensional (2D) cephalometric imaging is limited in its ability to accurately represent complex anatomical structures owing to distortion, magnification errors, and superimposition of bilateral structures. To overcome these limitations, three-dimensional (3D) imaging technologies have been introduced, among which cone-beam computed tomography (CBCT) has gained prominence [7]. CBCT provides high-resolution, 3D visualization of the craniofacial anatomy with significantly lower radiation exposure than conventional CT [8]. Its ability to render volumetric data in multiple planes allows for the precise assessment of anatomical landmarks, making it an invaluable tool in modern orthodontics and craniofacial research.

Mandibular morphology is not only influenced by growth patterns, but also by age, sex, and dental status [5]. Key anatomical landmarks such as the gonial angle, condylar inclination, sigmoid notch, and mandibular symphysis undergo continuous morphological changes over time [4]. While previous studies have examined these features in isolation or within limited sample groups [4,5], there remains a scarcity of comprehensive studies comparing mandibular morphology across different facial types using CBCT.

The primary aim of this study was to compare mandibular morphology among individuals with different vertical facial growth patterns, specifically normodivergent, hypodivergent, and hyperdivergent types, using CBCT. The objectives of this study included a detailed evaluation of mandibular features, such as the gonial angle, condylar inclination, mandibular notch depth, and symphysis morphology, in each facial type. Furthermore, this study aimed to make direct comparisons between the mandibular structures of normodivergent and hypodivergent individuals, normodivergent and hyperdivergent individuals, and hypodivergent and hyperdivergent individuals.

## Materials and methods

This cross-sectional observational study was conducted in the Department of Orthodontics and Dentofacial Orthopaedics at Kothiwal Dental College and Research Centre, Moradabad, Uttar Pradesh, India, from June 2023 to January 2025. Prior to commencement, ethical clearance was obtained from the Institutional Ethics and Review Board (IERB) under approval number KDCRC/IERB/04/2023/27. All subjects included in the study provided informed written consent for the use of their diagnostic data for research purposes. This study was conducted in accordance with the principles of the Declaration of Helsinki.

The sample size was calculated using G*Power software (version 3.1.9.2; Heinrich-Heine-Universität Düsseldorf, Düsseldorf, Germany). With a calculated effect size of 0.673, significance level of 5%, and study power of 80%, the minimum required sample size was determined to be 27, with nine subjects assigned to each group representing different vertical facial patterns.

Participants were selected from among patients who sought orthodontic treatment at the department. Subjects were required to be between 18 and 36 years of age, to have a skeletal Class I pattern with Angle’s Class I molar relationship, normal overjet and overbite, and a pleasant facial profile. Individuals with a history of orthodontic treatment, facial asymmetry, prior facial trauma or surgery, temporomandibular joint pain or sounds, pregnant and lactating females, and mandibular deviation during mouth opening were excluded from the study.

The selected subjects were grouped into normodivergent, hypodivergent, and hyperdivergent facial types based on Jarabak’s ratio [9]. Subjects with a Jarabak’s ratio between 62% and 65% were considered normodivergent, those with a ratio less than 62% were classified as hypodivergent, and those with a ratio greater than 65% were categorized as hyperdivergent [9]. Each group consisted of nine participants, forming a total of 27 samples.

CBCT was used to analyze the mandibular morphology. The imaging was performed using a CBCT machine (Carestream Dental LLC, Atlanta, Georgia, USA). Diagnostic records, including pre-treatment lateral cephalograms and CBCT scans, were used for all selected patients. Image acquisition was performed by an experienced radiologist at the Department of Oral Medicine and Radiology. Patients were prepared for scanning by removing all metallic objects, after which they were positioned in the scanner using a head restraint and aligned along the Frankfort Horizontal plane (a plane joining the lower border of the orbit and the uppermost point on the external auditory meatus) using a laser guide. Appropriate radiation protection measures were observed throughout the study.

CBCT scanning was performed using the following parameters: field of view (FOV), 8 × 10 cm; peak kilovoltage, 90 kVp; current, 4.0 mA current, voxel size, 180 µm; and exposure time, 8 s. The CBCT machine employed a cone-shaped beam for data acquisition, which was then reconstructed into volumetric images using the Feldkamp (Davis) Kress algorithm. The images were saved in Digital Imaging and Communications in Medicine (DICOM) format and reconstructed into 0.76 µm thick continuous slices using Carestream 3D Imaging Software.

**Figure 1:**
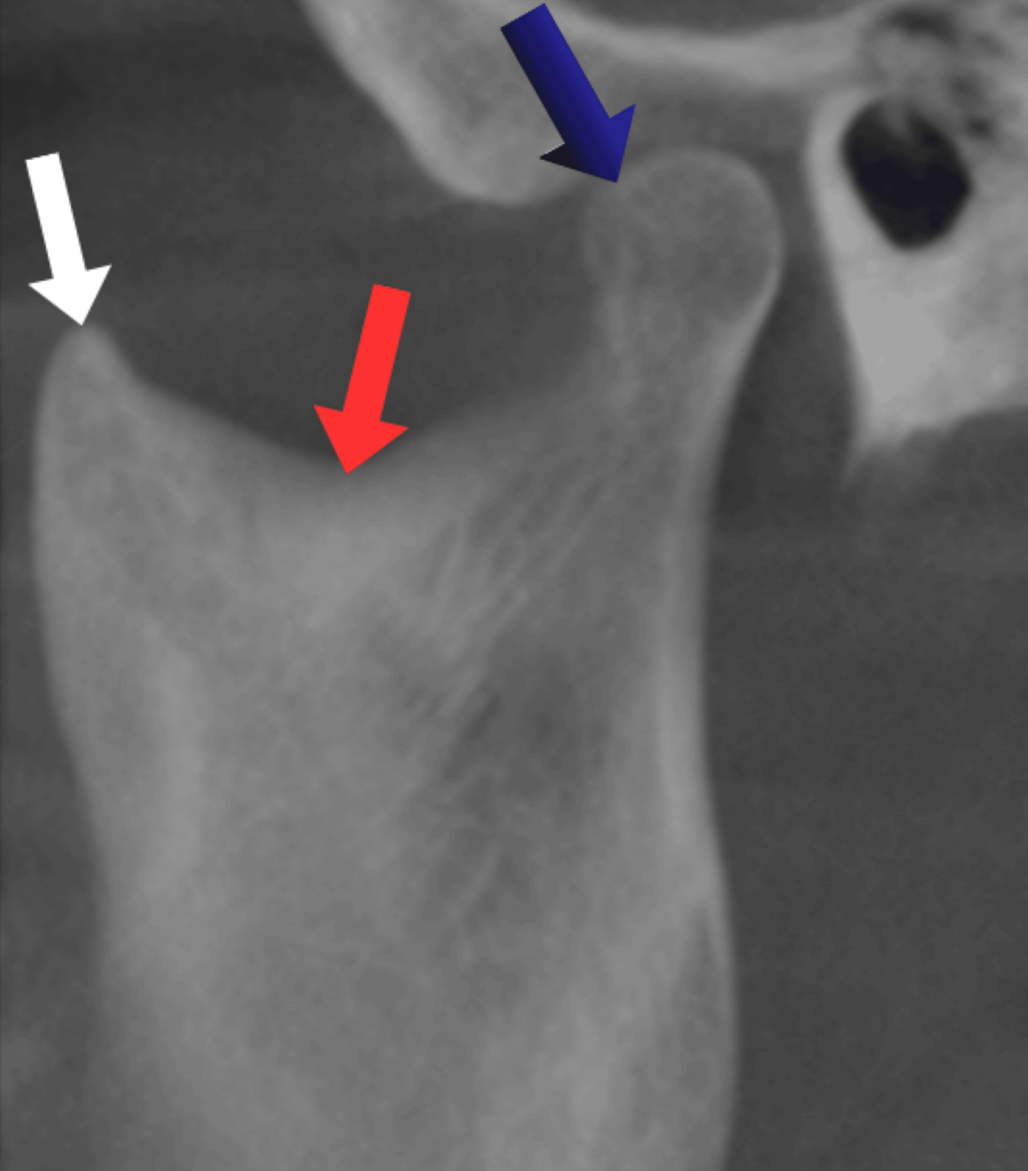
Morphology of coronoid (white arrow), condyle (blue arrow), and mandibular notch (red arrow) as assessed in sagittal view of CBCT. This figure depicts cone-beam computed tomographic image of a patient included in this study and used with the patients' permission. CBCT, cone-beam computed tomography

For radiographic assessment, CBCT images were oriented in all three planes of space to minimize measurement errors due to non-standard head posture. The analysis included the assessment of mandibular morphology, such as condylar width, length, height, and shape, coronoid process shape, mandibular (sigmoid) notch morphology, ramus height and width, and symphyseal height and thickness (Figure 1). The landmarks and measurements were standardized based on validated protocols and anatomical definitions from previous studies [9-13].

**Figure 2:**
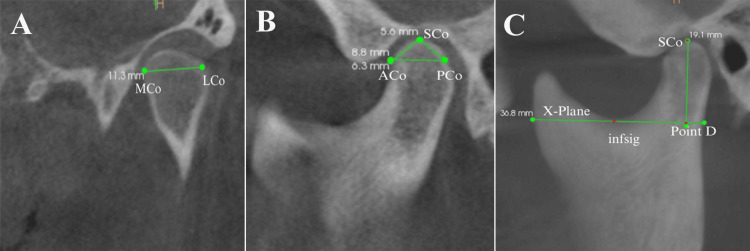
Condylar measurements in mm: (A) Width - measured in the coronal plane as the linear distance between the most medial mandibular condylar point (MCo) and the most lateral mandibular condylar point (LCo). (B) Length - measured in the sagittal plane as the distance between the anterior (ACo) and posterior (PCo) mandibular condylar points, which were identified as 4 mm inferior to the superior condylar point (SCo). (C) Height - measured as the vertical distance from the intersection of a horizontal tangent drawn through the most inferior point of the sigmoid notch (infsig) to the posterior border of the ramus, up to the superior-most point of the condyle (SCo). This figure depicts cone-beam computed tomographic images of a patient included in this study and used with the patients' permission.

Condylar morphology was evaluated by analyzing its width, length, height, and shape [10]. The condylar width was measured in the coronal plane as the linear distance between the most medial mandibular condylar point (MCo) and the most lateral mandibular condylar point (LCo). To determine the condylar length, the anterior (ACo) and posterior (PCo) mandibular condylar points were identified as 4 mm inferior to the superior condylar point (SCo), and the distance between them was measured in the sagittal plane. For the condylar height assessment, a horizontal tangent was drawn through the most inferior point of the sigmoid notch (infsig), and its intersection with the posterior border of the ramus was used as a reference. The vertical distance from this intersection to the superior-most point of the condyle (SCo) was measured to determine the height (Figure 2). The condylar shape was categorized according to the classification given in previous studies [11,12] into four distinct types: rounded, flat, beak-like, and concave, as visualized in the sagittal view of the CBCT scans (Figure 1).

Coronoid morphology was evaluated based on the shape of the coronoid process [11,12] (Figure 1). They were classified into two types: triangular and round, according to the visual appearance of the sagittal views of the CBCT images.

The mandibular notch morphology was similarly assessed using CBCT imaging (Figure 1). The morphological classification of the notch was performed by observing its shape and categorizing it into either a triangular or round type, based on the standards proposed in the literature [11,12].

**Figure 3:**
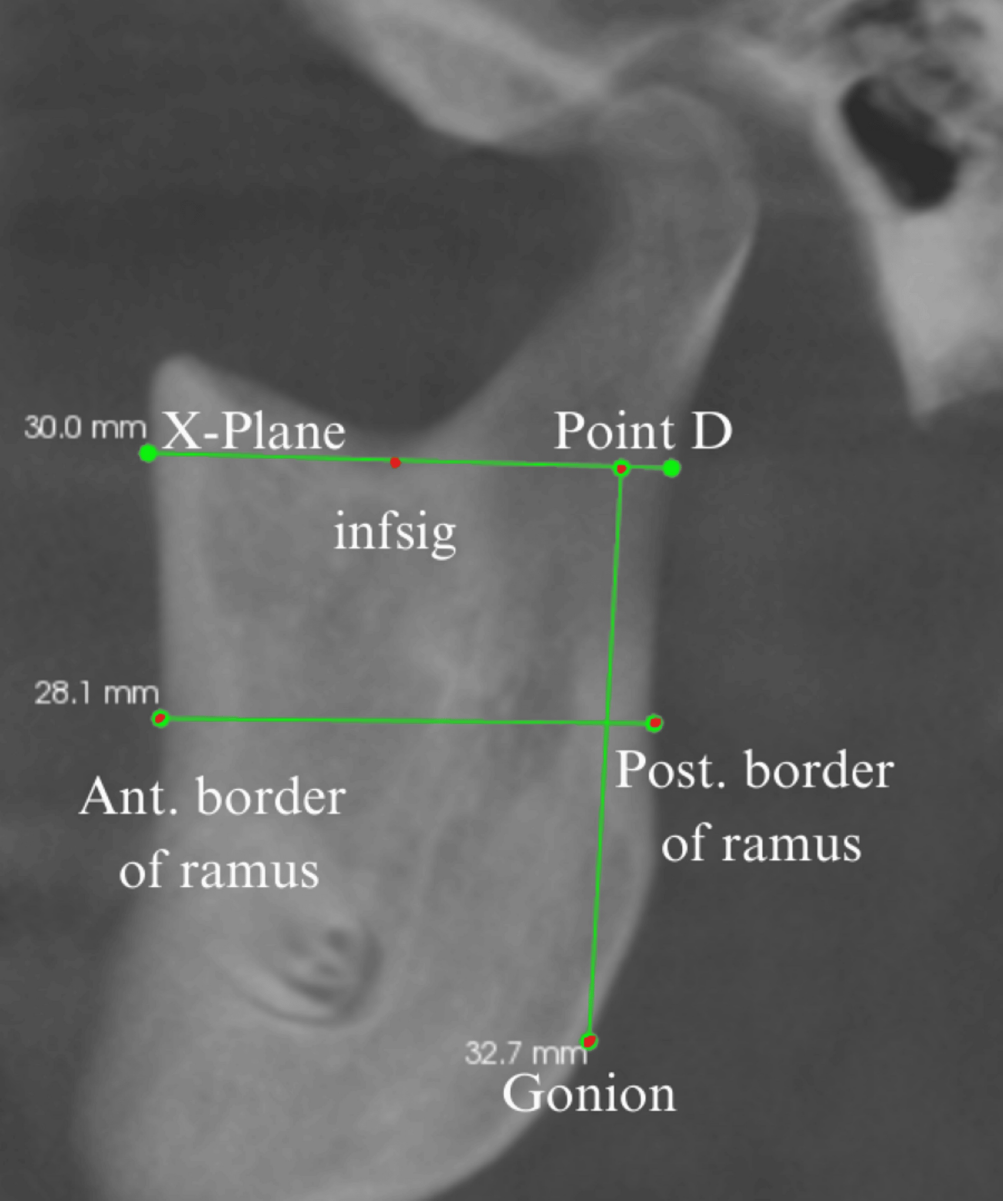
Ramus morphology measurements in mm, assessed in the sagittal view of CBCT. Ramal height was determined by drawing a tangent line from the infsig point (X-plane), parallel to the true horizontal reference, and measuring the vertical distance from point D on this line to the gonion. Ramus width was recorded as the distance between the anterior (Ant.) and posterior (Post.) borders of the ramus at the level of the occlusal plane. This figure depicts cone-beam computed tomographic image of a patient included in this study and used with the patients' permission.

Ramal morphology was evaluated by assessing the height and width of the mandibular ramus [9]. Ramal height was determined by drawing a tangent line from the infsig point (X-Plane), parallel to the true horizontal reference, and measuring the vertical distance from point D on this line to the gonion. Ramus width was recorded as the distance between the anterior and posterior borders of the ramus at the level of the occlusal plane (Figure 3).

**Figure 4:**
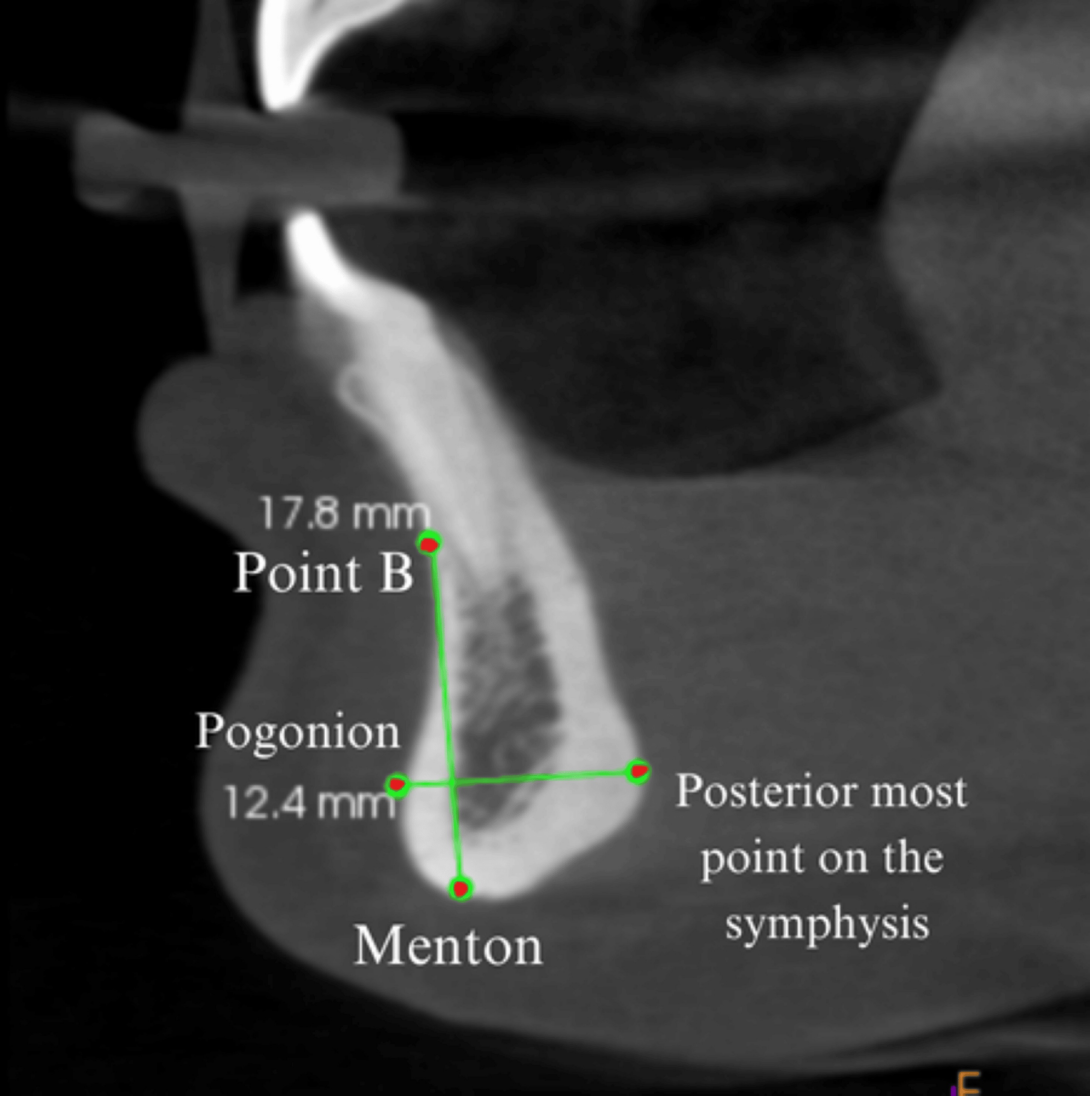
Mandibular symphysis measurements in mm. The symphyseal height was measured as the linear distance from point B to the menton. The maximum thickness was recorded as the horizontal distance between the pogonion and the most posterior point on the symphysis as assessed on the sagittal view of the CBCT scans. CBCT, cone-beam computed tomography This figure depicts cone-beam computed tomographic image of a patient included in this study and used with the patients' permission.

Symphyseal morphology was studied by measuring both the height and maximum thickness of the mandibular symphysis [13]. The symphyseal height was defined as the linear distance from the most superior point on the alveolar bone (point B) to the lowest point on the mandibular symphysis (menton). The maximum thickness was recorded as the horizontal distance between the pogonion and the most posterior point on the symphysis and assessed on the sagittal view of the CBCT scans (Figure 4).

All measurements were performed by the same examiner to minimize interobserver variability. Intra-examiner reliability was evaluated by repeating measurements for 10 randomly selected samples after a two-week interval. The intraclass correlation coefficient (ICC) for continuous variables ranged between 0.84 and 0.94, indicating excellent reliability, while kappa values for categorical data ranged between 0.92 and 0.94, confirming near-perfect agreement.

Statistical analysis was performed using the SPSS software version 21 (IBM Corp., Armonk, New York, USA). Descriptive statistics were calculated for all variables, and data normality was assessed using the Shapiro-Wilk test. Parametric tests were applied when the data followed a normal distribution. Statistical significance was set at p < 0.05.

## Results

The study involved 27 individuals, evenly divided into three groups: normodivergent, hypodivergent, and hyperdivergent, based on growth patterns, with each group comprising nine individuals. The majority of individuals were females in all groups. Despite this sex imbalance, the statistical analysis showed no significant association between sex and growth patterns, indicating an even sex distribution across the three groups (Table 1).

**Table 1:** Distribution of sex in study groups. Data presented in the form of frequency (n) and percentage (%); p-value > 0.05: not statistically significant.

Groups	Sex		Chi-square	p-value
	Female, n (%)	Male, n (%)		
Average grower	8 (29.63)	1 (3.70)	2.48	0.288
Horizontal grower	5 (18.52)	4 (14.82)		
Vertical grower	6 (22.22)	3 (11.11)		

In the assessment of condylar morphology, Type 4 condyles were the most frequently observed shape overall, particularly in individuals with hyperdivergent growth patterns. Types 1 and 2 were the least prevalent among all the groups. However, this distribution did not reach statistical significance, suggesting no meaningful relationship between the condylar shape and growth pattern classification (Table 2).

**Table 2:** Association of condylar shape with study groups using chi-square test of association. Data presented in the form of frequency (n) and percentage (%); p-value > 0.05: not statistically significant.

Groups	Condylar shape				Chi-square	p-value
	Type 1, n (%)	Type 2, n (%)	Type 3, n (%)	Type 4, n (%)		
Average grower	2 (7.41)	0 (0.00)	5 (18.52)	2 (7.41)	12.01	0.062
Horizontal grower	0 (0.00)	3 (11.11)	2 (7.41)	4 (14.82)		
Vertical grower	1 (3.70)	0 (0.00)	2 (7.41)	6 (22.22)		

Similarly, the coronoid process predominantly exhibited a Type 1 morphology in all three groups, especially in the hyperdivergent group, where it was exclusively present. This finding did not show a significant association with the growth patterns (Table 3).

**Table 3:** Association of coronoid shape with study groups using chi-square test of association. Data presented in the form of frequency (n) and percentage (%); p-value > 0.05: not statistically significant.

Groups	Coronoid shape		Chi-square	p-value
	Type 1, n (%)	Type 2, n (%)		
Average grower	6 (22.22)	3 (11.11)	3.43	0.179
Horizontal grower	7 (25.93)	2 (7.41)		
Vertical grower	9 (33.33)	0 (0.00)		

The morphology of the mandibular notch showed a nearly even split between the two observed types. The distribution appeared random, and no statistically significant correlation was found between the notch shape and vertical facial pattern (Table 4).

**Table 4:** Association of mandibular notch shape with study groups using chi-square test of association. Data presented in the form of frequency (n) and percentage (%); p-value > 0.05: not statistically significant.

Groups	Mandibular notch shape		Chi-square	p-value
	Type 1, n (%)	Type 2, n (%)		
Average grower	4 (14.82)	5 (18.52)	0.29	0.862
Horizontal grower	5 (18.52)	4 (14.82)		
Vertical grower	4 (14.82)	5 (18.52)		

When analyzing linear measurements of the mandible, it was noted that the normodivergent group exhibited the greatest condylar width, whereas the hypodivergent group demonstrated the highest condylar height. The condylar length remained relatively uniform across all three groups. In the ramus region, the normodivergent group showed the greatest ramus height, whereas the hypodivergent group exhibited the widest ramus. Conversely, the hyperdivergent group presented with a relatively taller but narrower ramus. In the symphysis region, hyperdivergent individuals displayed the highest mean symphysis height, whereas hypodivergent individuals displayed the thickest symphysis. Normodivergent participants had balanced values for both the height and thickness of the symphysis (Table 5).

**Table 5:** Descriptive analysis of dependent variables for study groups. Data presented as mean and standard deviation. n, number of samples in the group

Parameters	Groups	n	Mean	Standard deviation	95% confidence interval of mean	
					Upper	Lower
Condylar width (mm)	Average	9	8.10	1.16	8.994	7.206
	Horizontal	9	8.01	1.84	9.422	6.578
	Vertical	9	7.65	1.36	8.708	6.603
Condylar length (mm)	Average	9	19.01	2.96	21.279	16.721
	Horizontal	9	19.23	3.86	22.207	16.260
	Vertical	9	18.02	3.17	20.464	15.581
Condylar height (mm)	Average	9	17.31	2.87	19.517	15.105
	Horizontal	9	21.18	3.60	23.963	18.415
	Vertical	9	18.83	3.03	21.168	16.499
Ramus height (mm)	Average	9	34.90	2.62	36.920	32.880
	Horizontal	9	32.70	6.89	38.001	27.399
	Vertical	9	33.48	5.59	37.786	29.191
Ramus width (mm)	Average	9	29.96	3.46	32.631	27.302
	Horizontal	9	32.03	3.61	34.815	29.251
	Vertical	9	26.91	3.42	29.543	24.279
Symphysis height (mm)	Average	9	16.60	2.32	18.388	14.812
	Horizontal	9	16.01	2.08	17.614	14.411
	Vertical	9	16.91	1.98	18.437	15.385
Symphysis thickness (mm)	Average	9	12.54	1.89	14.001	11.089
	Horizontal	9	13.03	2.14	14.684	11.383
	Vertical	9	10.67	1.64	11.940	9.415

Statistical analysis using one-way analysis of variance (ANOVA) revealed significant differences in ramus width and symphysis thickness among the groups, with p-values of 0.017 and 0.036, respectively. No statistically significant differences were observed in other anatomical parameters (Table 6).

**Table 6:** Comparison of study groups for different variables by one-way ANOVA test. *p-value < 0.05: significant. ANOVA, analysis of variance

Parameters	Sum of squares	Mean square	F-value	p-value
Condylar length (mm)	7.43	3.71	0.329	0.723
Condylar width (mm)	0.97	0.48	0.221	0.803
Condylar height (mm)	68.70	34.35	3.381	0.051
Ramus height (mm)	22.36	11.18	0.391	0.680
Ramus width (mm)	119.53	59.76	4.867	0.017*
Symphysis height (mm)	3.75	1.87	0.411	0.668
Symphysis thickness (mm)	27.81	13.90	3.831	0.036*

Further post-hoc testing using Tukey’s test revealed that significant differences were specifically between the hypodivergent and hyperdivergent groups. By contrast, comparisons involving the normodivergent group did not yield statistically significant differences. Although most anatomical measurements showed minor variations across groups, individuals with hyperdivergent growth patterns exhibited greater mean deviations in condylar height, ramus width, and symphysis height, suggesting a trend toward more pronounced morphological divergence in these parameters (Table 7).

**Table 7:** Post-hoc analysis by Tukey's test for variables such as ramus width and symphysis thickness. *p-value < 0.05: significant.

Pairwise groups		Mean difference	Standard error	t-value	p-value
Post-hoc comparisons for ramus width					
Average vs.	Horizontal	-2.06	1.65	-1.251	0.436
	Vertical	3.05	1.65	1.850	0.175
Horizontal vs.	Vertical	5.12	1.65	3.101	0.013*
Post-hoc comparisons for symphysis thickness					
Average vs.	Horizontal	-0.48	0.89	-0.544	0.850
	Vertical	1.86	0.89	2.078	0.116
Horizontal vs.	Vertical	2.35	0.89	2.622	0.038*

Spearman correlation analysis between the mandibular notch type and various mandibular measurements revealed predominantly weak and inconsistent associations across the different growth pattern groups. In the overall sample, the strongest negative correlation was observed with symphysis height (r = -0.52), suggesting that as the symphysis height increases, the likelihood of a certain mandibular notch type may decrease. Among hyperdivergent individuals, condylar height showed a moderately strong negative correlation (r = -0.69), indicating that taller condyles were associated with a specific notch type in this group. Additionally, moderate negative correlations were noted between the mandibular notch type and both ramus width (r = -0.52) and condylar height (r = -0.34) in the hypodivergent group. However, most of the correlations across all groups were weak (r values close to 0), implying little to no meaningful relationship between the mandibular notch type and most of the assessed anatomical variables. Overall, these findings suggested that mandibular notch morphology was not strongly influenced by variations in mandibular dimensions across different growth patterns (Table 8).

**Table 8:** Spearman correlation (r-value) between mandibular notch type and other variables. *p-value < 0.05: significant.

Variables	Average grower		Horizontal grower		Vertical grower	
	r-value	p-value	r-value	p-value	r-value	p-value
Condylar width	-0.08	0.056	-0.17	0.071	-0.08	0.081
Condylar length	0.01	0.034*	-0.08	0.078	-0.08	0.067
Condylar height	-0.26	0.001*	-0.34	0.002*	-0.69	0.002*
Ramus height	0.01	0.004*	-0.17	0.003*	0.34	0.001*
Ramus width	-0.04	0.001*	-0.52	0.001*	-0.56	0.001*
Symphysis height	-0.52	0.001*	-0.21	0.001*	0.21	0.001*
Symphysis thickness	0.17	0.072	-0.26	0.001*	-0.04	0.056

## Discussion

Understanding the intricacies of facial morphology and developmental trends is essential for orthodontic practitioners to tailor therapeutic interventions. This study aimed to assess alterations in mandibular morphology linked to hypodivergent, hyperdivergent, and normodivergent growth patterns using CBCT. In terms of condylar morphology, the predominance of Type 4 shapes (concave) across all groups, particularly within the hyperdivergent group, and Type 3 shapes (beak type) in the normodivergent group suggested a potential morphological trend, albeit not statistically significant. The reduced prevalence of Type 1 and Type 2 condyles indicated that these shapes might be less common in the general population. Previous studies have shown that condylar morphology can reflect functional adaptation in response to biomechanical loading, which varies among different facial patterns [14]. Hyperdivergent individuals often exhibit reduced masticatory forces, potentially leading to adaptive changes in condylar shape. However, the lack of significant correlation in this study implies that while trends are observable, they may not be robust enough to serve as diagnostic indicators on their own.

Similarly, the coronoid process most commonly displayed a Type 1 morphology (triangular), particularly in the hyperdivergent group, where it was exclusively present. While this observation may suggest a relationship between the vertical growth pattern and coronoid shape, the absence of statistical significance reduces its clinical applicability. This aligns with the conclusions drawn in earlier investigations, where the predominant coronoid morphology was triangular (Type 1) [15,16]. The coronoid process plays a key role in temporalis muscle attachment, and its morphology may reflect muscular activity patterns, which tend to differ with facial divergence. More vertical growth patterns associated with hyperdivergence might involve lower masticatory muscle activity, leading to less morphological variation in the coronoid region [17]. However, this assumption warrants further investigation using a larger, more balanced sample.

Mandibular or sigmoid notch morphology, evaluated as being of two types, showed an almost equal distribution across all groups, with no significant association with the growth pattern. This finding supports the hypothesis that mandibular notch morphology may be more genetically determined or developmentally stable and less affected by functional or skeletal factors. The morphology of the structure is predominantly influenced by the morphology of the coronoid and condylar processes [18]. In the current study, no noteworthy correlation was identified between the morphology of the coronoid and condylar processes and facial patterns, which may have contributed to the lack of a significant association between the morphology of the mandibular notch and facial patterns in our analysis. This neutrality in distribution emphasizes that notch morphology, unlike condyle or ramus, may not serve as a reliable marker for assessing skeletal divergence.

Linear measurements of mandibular structures revealed intriguing patterns of variation across the groups. Normodivergent individuals had the widest condyles and the tallest rami, suggesting a balanced vertical and horizontal growth trajectory. Hypodivergent participants, characterized by shorter facial heights and stronger muscle function, exhibited the highest condylar height and greatest ramus width, reinforcing the existing literature that associates low-angle skeletal patterns with increased bone mass and muscular influence [4]. In contrast, hyperdivergent individuals, typically marked by longer lower facial height and weaker musculature, showed taller but narrower rami, as well as the greatest symphysis height [4]. Singer et al. [19] reported a reduction in ramus height among subjects exhibiting vertical growth patterns and the presence of a deep mandibular notch. Mangla et al. [4] determined that ramus height is elevated in hypodivergent individuals relative to their normodivergent and hyperdivergent counterparts. Conversely, the present study revealed that ramus height was more pronounced in normodivergent individuals than in those with hypodivergent or hyperdivergent growth patterns.

The present study showed that Class I hyperdivergent individuals showed increased symphysis height, which was attributed to vertical mandibular growth, while hypodivergent individuals exhibited reduced symphysis height due to a horizontal growth pattern that limited vertical elongation. Meredith [20] observed that the progeny of parents exhibiting increased symphyseal height tended to possess a thicker symphysis. Aki et al. [6] identified that a mandible characterized by anterior growth orientation was correlated with reduced height, increased depth, diminished ratio, and an expanded angle of the symphysis. These findings were corroborated by the results of the current study. In contrast, a posterior growth orientation was linked to increased height, reduced depth, elevated ratio, and diminished symphysis angle. Chen et al. [21] reported that symphysis height in both male and female subjects was markedly greater in the Class II hyperdivergent cohort than in the Class I hyperdivergent cohort.

From a clinical perspective, the results of this study imply that while some mandibular parameters, such as ramus width and symphysis thickness, vary significantly between growth patterns and may assist in diagnostic or orthodontic planning, others, such as condylar and mandibular notch morphology, may have limited utility as standalone indicators. Instead, a comprehensive assessment, involving both qualitative and quantitative features, is essential for evaluating vertical growth patterns.

One of the key strengths of this study was the use of CBCT, which provided high-resolution 3D imaging that enabled a detailed evaluation of mandibular morphology. This advanced imaging technique allows for the precise measurement of a wide range of mandibular parameters, supporting a comprehensive analysis of structural variations across different vertical facial growth patterns. The study incorporated both qualitative assessments, such as classifications of condylar, coronoid, and mandibular notch morphologies, and quantitative linear measurements of structures, such as the ramus and symphysis. As a result, this study offered an in-depth understanding of how mandibular components differed among normodivergent, hypodivergent, and hyperdivergent individuals.

Despite these strengths, this study has several limitations. The age range of the participants was restricted to 18-36 years, which limited insight into developmental and age-related changes in mandibular morphology. Including a broader age range may reveal important trends across different life stages. Although females were the majority in all groups, the study did not conduct a detailed analysis of sex-related morphological differences, which may have influenced the outcomes owing to underlying genetic and hormonal factors. The small sample size (n = 27) reduced the statistical power and limited the generalizability of the results. Additionally, the cross-sectional design captured data at a single point in time, preventing the observation of longitudinal changes in mandibular structures. Finally, condylar morphology was assessed only on the left side, which may not accurately represent possible bilateral asymmetries in condylar development or function.

## Conclusions

The present study demonstrated that certain morphological features of the mandible, such as ramus width and symphysis thickness, vary significantly among individuals with different vertical facial growth patterns. Hyperdivergent individuals tended to exhibit greater morphological divergence, particularly in terms of condylar height, ramus width, and symphysis height. Although the qualitative assessment of condylar, coronoid, and mandibular notch morphologies revealed observable patterns, these did not reach statistical significance in relation to the growth patterns. Furthermore, the mandibular notch type showed predominantly weak correlations with other mandibular parameters across all groups, indicating a limited predictive value. Overall, the findings suggested that while some mandibular characteristics were influenced by vertical growth patterns, many aspects of mandibular morphology remained relatively stable. This underscores the complexity of craniofacial development and highlights the need for larger, longitudinal studies to better understand these relationships.
